# Supplementary material for: Process Evaluation of a Participative Organizational Intervention as a Stress Preventive Intervention for Employees in Swedish Primary Health Care
Source: Int J Environ Res Public Health. 2020 Oct 6;17(19):7285. doi: 10.3390/ijerph17197285 (PMC7579215; doi:10.3390/ijerph17197285)
Supplement: Supplementary file 1 [file ijerph-17-07285-s001.zip › Appendix 1.docx]

Appendix 1. ProMES Fidelity Checklists, adapted from ProMES Meta-Analysis Questionnaire [[1](#_ENREF_1)] with permission from Robert D. Pritchard

**Part I: A checklist for starting a ProMES project: general**

|  | **Yes** | **No** | **Don`t know** | **Additional information (for example: when, how, how many times, how much, what level, and so on)** |
| --- | --- | --- | --- | --- |
| Facilitator completely familiar with the background and logic of ProMES |  |  |  |  |
| Benefits and costs clearly explained to all unit personnel |  |  |  | When: |
| Benefits and costs clearly explained to supervisor |  |  |  | When: |
| Benefits and costs clearly explained to higher level management. |  |  |  | When |
| Benefits and costs clearly explained to top level management. |  |  |  | When |
| Support exists at all levels between highest management level and the unit |  |  |  | Missing level: |
| Public management support on a regular basis throughout project planned |  |  |  | When, How often: |
| Commitment by the management that the work technology will be stable during the project. (development and at least 6 months of feedback implementation). |  |  |  |  |
| Commitment by the management that organizational structure will be stable during the project (development and at least 6 months of feedback implementation). |  |  |  |  |
| Management has dealt with issues expected to come up: job loss if productivity goes up |  |  |  | Details: |
| Management has dealt with issues expected to come up: compensation if productivity goes up |  |  |  | Details: |
| Management has dealt with issues expected to come up: how productivity measurement will be tied to pay |  |  |  | Details: |
| ProMES will be used as the new method of evaluating the unit |  |  |  |  |
| Management committed to help in solving project problems |  |  |  |  |

**Part II: A checklist for starting a ProMES project: resources needed for development and implementation:**

|  | **Yes** | **No** | **Don`t know** | **Additional information (for example: when, how, how many times, how much, what level, and so on)** |
| --- | --- | --- | --- | --- |
| Time for design team meetings planned |  |  |  | Frequency and duration: |
| A setting provided for uninterrupted design team meetings |  |  |  |  |
| Full attendance by design team at design meetings enabled |  |  |  |  |
| 60-70 hours facilitator time outside meetings admitted |  |  |  |  |
| Access to and collection of existing organizational data possible |  |  |  |  |
| Resources will be provided for training for unit members on how to  use the feedback reports, |  |  |  |  |
| Resources will be provided for training for supervisors and  managers on how to use the feedback reports |  |  |  |  |
| Resources will be provided for preparation and distribution of feedback reports |  |  |  |  |
| Regular meetings of the unit members to discuss feedback reports  possible (1hour per feedback period) |  |  |  |  |
| Decide what software will be used to give feedback |  |  |  | Software: |
| Resources for training in use of software provided |  |  |  |  |
| Decide when and how the organization will take over preparation and  distribution of the feedback reports |  |  |  |  |

**Part III: A checklist for a ProMES project at the time for the first feedback report**

**1. To what extent were there serious problems in the target unit at the start of the project?**  Examples would include serious conflicts within the group or with the group and management, major organization problems, serious management problems, etc.

___ 5. There were many serious problems

___ 4.

___ 3. There were some moderately serious problems

___ 2.

___ 1. There were no meaningful problems

**2. Degree of trust between target unit members and management.**

1. Degree of trust the target unit has in management

___ 5. Very much. Members of the target unit felt that management would never take advantage of them.

___ 4.

___ 3. Moderate. Members of the target unit trusted management would be supportive in most situations but felt they would take advantage of them occasionally.

___ 2.

___ 1. Very little. Target unit members felt that management would take advantage of them at every opportunity.

**3. Degree of trust management had in the members of the target unit.**

___ 5. Very much. Management felt that the target unit would never take advantage of them.

___ 4.

___ 3. Moderate. Management felt that the target unit would be supportive in most situations but felt that they would take advantage of them occasionally.

___ 2.

___ 1. Very little. Management felt that the target unit would take advantage of them at every opportunity.

**4. Highest organization level where the ProMES project was supported**: (Check the highest level.)

___ 5. Top management: parent. The highest levels of the parent organization’s management directly supported the project. (If there is no parent organization and the top level of the local organization supported the project, use this rating.)

___ 4. Top management: local. The highest levels of the local organization’s management directly supported the project, but not the top level of the parent organization.

___ 3. Middle management: local. Middle management of the local organization directly supported the project, but not top management.

___ 2. Lower level management: local. Lower level management of the local organization directly supported the project, but not middle or top management.

___ 1. Supervisors only. The project was supported at the supervisory level but not by any levels of management.

**6. Was the setting in which the design teams met to develop the system conducive to doing work?** (For example, a setting might be too noisy, too hot or cold, or have too many distractions.)

___ 5. Setting was conducive to doing work in all aspects.

___ 4.

___ 3. Setting had some characteristics that made working somewhat more difficult.

___ 2.

___ 1. Setting possessed elements which made it very difficult to work.

**12. Amount of consensus reached on the major issues**:

___ 5. Complete agreement was reached on all major issues

___ 4. Clear consensus was reached on all major issues

___ 3. Clear consensus was reached on most major issues, but not all

___ 2. Clear consensus was reached on some major issues, but not most of them

___ 1. Clear consensus was reached on only a few major issues

**13. Unit personnel in the design team were actively involved in design team meetings?** (Actively involved means they were present, attended carefully to what was happening, clearly understood what was going on, and spoke regularly.)

___ 5. I strongly agree.

___ 4

___ 3 I neither agree or disagree

___ 2

___ 1 I strongly disagree

**14. Tolerance for minority opinions.** To what extent were positions different from the majority position tolerated by the design team?

___ 5. Different positions were highly valued.

___ 4. Different positions were somewhat valued.

___ 3. Different positions were tolerated.

___ 2. Different positions were somewhat discouraged.

___ 1. Different positions were not tolerated.

**15. The amount of influence the supervisor(s) had on the content of the completed system:**

___ 5. System development was dominated by the supervisor(s).

___ 4. The supervisor(s) had more influence than the average person in the group.

___ 3. The supervisor(s) had about the same influence as the average person in the group.

___ 2. The supervisor(s) had less influence than the average person in the group.

___ 1. The supervisor(s) had no influence on the content of the completed system.

**16. During the development of the system, how were the members of the target unit who were not part of the design team informed about the design team's activities and progress?**

_____ 5. A formal process was used and done on a regular basis

_____ 4.

_____ 3. A formal process was used (such as memos, announcements, rotating personnel through the design team, etc.) but this was not done on a regular basis

_____ 2.

_____ 1. No formal process, but it is assumed the rest of the target unit was informed informally by design team members.

_____ The entire unit was in the design team, so it was not an issue.

**17. Measurement system seems to be accepted by unit personnel.**

___ 5. I strongly agree.

___ 4

___ 3 I neither agree or disagree

___ 2

___ 1 I strongly disagree

**18. Measures used are under the control of the unit personnel.**

___ 5. I strongly agree.

___ 4

___ 3 I neither agree or disagree

___ 2

___ 1 I strongly disagree

Please **check all that apply:**

______Objectives are stated in clear terms.

______Objectives are designed so that if exactly that objective was done, the organization would benefit.

______Set of objectives covers all important aspects of the work.

______Objectives are consistent with the objectives of the broader organization.

______Higher management is committed to each objective.

______The number of objectives is manageable (normally 4 to 8).

Please **check all that apply:**

______Indicators are consistent with the objectives of the broader organization

______Indicators are valid measures of objectives

______All important aspects of each objective is covered by the set of indicators

______Higher management is committed to the indicators

______Indicators are largely under the control of unit personnel

______Indicators are understandable and meaningful to unit personnel

______It is possible to provide information on the indicator in a timely manner

______Accurate indicator data are cost-effective to collect

______The information provided by the indicator are neither too general nor too specific.

(Validity. A test of the validity of an indicator can be made by answering two questions. First, "If the unit was very high on the measure, would this be good for the organization? A second and related question to ask is "What are the long-term implications of the unit making such an index look good?")

**Included in Indicator Information Form: (**Please check all that apply)

______Objective number and name

______Short indicator name

______Rationale including how indicator is related to overall organizational objectives

______How exactly is indicator calculated

______Data source for indicator

______Who is responsible for gathering indicator data and

______Who is responsisble for entering data into report program

**19 What type of training did members of the target unit receive to help them read and interpret the data in the feedback report?**

___ 5. The system was explained in great detail to the target unit and they were given examples of feedback data and how they would be used. (Use this response if the entire unit was on the design team.)

___ 4.

___ 3. The system and how it worked was explained to the entire unit in a meeting or other formal way.

___ 2.

___ 1. No formal training was done other than the design team informally explaining the system to their peers.

**20 What type of training did supervisors receive to help them read and interpret the data in the feedback report?**

___ 5. The system was explained in great detail to the supervisors and they were given examples of feedback data and how they would be used. (Use this response if the entire unit was on the design team.)

___ 4.

___ 3. The system and how it worked was explained to the supervisors in a meeting or other formal way.

___ 2.

___ 1. No formal training was done other than design team informally explaining the system to their supervisors.

**21. What type of training did management receive to help them read and interpret the data in the feedback report?**

___ 5. The system was explained in great detail to management and they were given examples of feedback data and how they would be used.

___ 4.

___ 3. The system and how it worked was explained to management in a meeting or other formal way.

___ 2.

___ 1. No formal training was done other than design team members informally explaining the system to management.

**22. At the start of the project (i.e., when the design team started meeting), to what extent did management support the project?**  Management support is composed of verbal support to the project directors and the target unit, support with organizational resources such as paid employee time and space to work, and publicly stated support of the project to others in the organization.

___ 5. High. Management was willing to invest as many resources and support as needed to insure the success of the project, and helped the project whenever help was needed.

___ 4.

___ 3. Moderate. Management was willing to invest some resources and support in the project, and was helpful in some instances and not in others.

___ 2.

___ 1. Low. Management was unwilling to invest any resources and support in the project, and was uncooperative with people involved with the project.

**The management approval step occurred:**

______when the objectives and indicators were finished

______when the contingencies were completed.

______Both when the objectives and indicators were finished and when the contingencies were completed.

Management was given written versions of the proposed objectives and indicators and given time prior to the meeting to study these documents.

______Yes

______No

______I do not know

**23. What percentage of the objectives (products) were *substantially* changed to obtain formal management approval? (A slight wording change, combining two products into one, or dividing a product into two products are not substantial changes. Adding a new product, dropping a product, or significantly altering the meaning of a product is a substantial change.) ______%**

**24. What percentage of the indicators were *substantially* changed to obtain formal approval? (Use the same idea for “substantial” as in changes of objectives above.) ______%**

**25. What percentage of the contingencies were *substantially* changed to obtain formal approval? (Substantial here means a change that alters the expected level or other effectiveness scores so that the contingency is really different than it was. A change of 3-5 effectiveness score points would not normally be considered a substantial change.) ______%**

1. Pritchard, R.D., et al., *The productivity measurement and enhancement system: a meta-analysis.* Journal of Applied Psychology, 2008. **93**(3): p. 540-67.
