# Supplementary material for: Process Evaluation of a Participative Organizational Intervention as a Stress Preventive Intervention for Employees in Swedish Primary Health Care
Source: Int J Environ Res Public Health. 2020 Oct 6;17(19):7285. doi: 10.3390/ijerph17197285 (PMC7579215; doi:10.3390/ijerph17197285)
Supplement: Supplementary file 1 [file ijerph-17-07285-s001.zip › Appendix 2.docx]

Appendix 2. Items in the process evaluation questionnaire.

|  | Respons¹, N (%) | | | | |
| --- | --- | --- | --- | --- | --- |
|  | 1 or 2 | 3 | 4 | 5 | 6 |
| ProMES is a method suitable for |  |  |  |  |  |
| Clarifying what is important. | - | 3 (6) | 22 (45) | 22 (45) | 2 (4) |
| Clarifying priorities. | - | 5 (10) | 22 (45) | 20 (41) | 2 (4) |
| Reducing unnecessary efforts | 3 (6) | 11 (23) | 21 (44) | 11 (23) | 2 (4) |
| Giving the management more control | 3 (6) | 9 (19) | 22 (45) | 7 (14) | 8 (16) |
| Giving employees more control | 3 (6) | 5 (11) | 18 (38) | 17 (36) | 4 (9) |
| Increasing employees' participation in decision making. | 2 (4) | 4 (8) | 22 (45) | 16 (33) | 5 (10) |
| Giving employees better feedback. | 2 (4) | 6 (12) | 22 (45) | 16 (33) | 3 (6) |
| Giving employees the chance to fix problems before they become serious. | 2 (4) | 2 (4) | 28 (57) | 14 (29) | 3 (6) |
| Giving employees an opportunity to improve things. | 1 (2) | 2 (4) | 20 (42) | 23 (48) | 2 (4) |
| Promes |  | | | | |
| Is time consuming to work with. | 4 (8) | 6 (12) | 27 (55) | 8 (17) | 4 (8) |
| Is difficult to understand. | 28 (57) | 6 (12) | 12 (25) | - | 3 (6) |
| Takes a long time to introduce. | 13 (26) | 14 (29) | 12 (25) | 7 (14) | 3 (6) |
| Is easy to use. | 4 (8) | 11 (23) | 22 (45) | 8 (16) | 4 (8) |
| To what extent do you find that the following is correct: |  | | | | |
| Enough information was provided for the work on the ProMES. | 1 (2) | 3 (6) | 22 (45) | 16 (33) | 7 (14) |
| The consultant provided enough support while we were working on the method. | - | 1 (2) | 18 (37) | 22 (45) | 8 (16) |
| The management provided enough support during the time we worked with the method | 2 (4) | 5 (10) | 20 (41) | 17 (35) | 5 (10) |
| The ProMES method is a good method to work with when it comes to increasing the efficiency of the work. | 1 (2) | 4 (8) | 30 (61) | 11(23) | 3 (6) |
| The ProMES method is a good method to work with when it comes to reducing my work-related stress. | 2 (4) | 11 (23) | 25 (51) | 8 (16) | 3 (6) |
| The method fits well with the unit's needs and working methods. | 1 (2) | 11 (22) | 23 (47) | 10 (21) | 4 (8) |
| I would like to continue working on the method. | 3 (6) | 12 (25) | 16 (33) | 11 (23) | 6 (13) |
| The method is easy to maintain. | 3 (6) | 11 (24) | 21 (46) | 5 (11) | 6 (13) |
|  |  |  | | | |
| If you summarize the entire work with the ProMES method, how satisfied are you?² | 2 (4) | 9 (19) | 26 (54) | 7 (15) | 4 (8) |

¹Answer alternatives: 1 = strongly disagree; 2 = disagree to some extent; 3 = neither agree nor disagree; 4 = agree to some extent; 5= strongly agree; 6 = I can´t take a position on this.

²Answer alternatives: from 1= very dissatisfied to 5 = very satisfied and 6 = I can´t take a position on this.
