# Supplementary material for: Process Evaluation of a Participative Organizational Intervention as a Stress Preventive Intervention for Employees in Swedish Primary Health Care
Source: Int J Environ Res Public Health. 2020 Oct 6;17(19):7285. doi: 10.3390/ijerph17197285 (PMC7579215; doi:10.3390/ijerph17197285)
Supplement: Supplementary file 1 [file ijerph-17-07285-s001.zip › Appendix 3.docx]

Appendix 3. Items in the process evaluation questionnaire in relation to taxonomies of Proctor (2011) and Linnan and Steckler (2002).

| Process evaluation question |  |
| --- | --- |
|  |  |
| ProMES is a method suitable for |  |
| Clarifying what is important. | Appropriateness/Usefulness |
| Clarifying priorities. | Appropriateness/Usefulness |
| Reducing unnecessary efforts | Appropriateness/Usefulness |
| Giving the management more control | Appropriateness/Usefulness |
| Giving employees more control | Appropriateness/Usefulness |
| Increasing employees' participation in decision making. | Appropriateness/Usefulness |
| Giving employees better feedback. | Appropriateness/Usefulness |
| Giving employees the chance to fix problems before they become serious. | Appropriateness/Usefulness |
| Giving employees an opportunity to improve things. | Appropriateness/Usefulness |
| Promes |  |
| Is time consuming to work with. | Acceptability |
| Is difficult to understand. | Acceptability |
| Takes a long time to introduce. | Acceptability |
| Is easy to use. | Acceptability |
| To what extent do you find that the following is correct: |  |
| Enough information was provided for the work on the ProMES. | Satisfaction (work procedures) |
| The consultant provided enough support while we were working on the method. | Satisfaction (delivery of the method) |
| The management provided enough support during the time we worked with the method | Satisfaction (delivery of the method) |
| The ProMES method is a good method to work with when it comes to increasing the efficiency of the work. | Satisfaction (content) |
| The ProMES method is a good method to work with when it comes to reducing my work-related stress. | Satisfaction (content) |
| The method fits well with the unit's needs and working methods. | Appropriateness |
| I would like to continue working on the method. | Sustainability |
| The method is easy to maintain. | Feasibility |
|  |  |
| If you summarize the entire work with the ProMES method, how satisfied are you?² | Satisfaction |
